# Prolactinomas: evolution after menopause

**DOI:** 10.1590/2359-3997000000138

**Published:** 2016-01-01

**Authors:** Maria Susana Mallea-Gil, Marcos Manavela, Analia Alfieri, Maria Carolina Ballarino, Alberto Chervin, Karina Danilowicz, Sabrina Diez, Patricia Fainstein Day, Natalia García-Basavilbaso, Mariela Glerean, Mirtha Guitelman, Débora Katz, Monica Graciela Loto, Marcela Martinez, Karina Miragaya, Daniel Moncet, Amelia Susana Rogozinski, Marisa Servidio, Graciela Stalldecker, Marcelo Vitale, Laura Boero

**Affiliations:** 1 Departamento de Neuroendocrinología (Neuroendocrinology Department) Sociedad Argentina de Endocrinología y Metabolismo Ciudad Autónoma de Buenos Aires Argentina Departamento de Neuroendocrinología (Neuroendocrinology Department), Sociedad Argentina de Endocrinología y Metabolismo, Ciudad Autónoma de Buenos Aires, Argentina

**Keywords:** Prolactinoma, menopause, dopamine agonist, bromocriptine, cabergoline

## Abstract

**Objetive:**

The aim was to assess the evolution of tumor size and prolactin (PRL) levels in patients with micro and macroprolactinomas diagnosed and treated with dopamine agonists during fertile age, and the effects of suspension of drugs after menopause. Retrospective study, 29 patients with prolactinomas, 22 microadenomas and 7 macroadenomas, diagnosed during their fertile age were studied in their menopause; treatment was stopped in this period. Age at menopause was 49 ± 3.6 years. The average time of treatment was 135 ± 79 months. The time of follow-up after treatment suspension was 4 to 192 months.

**Results:**

Pre-treatment PRL levels in micro and macroadenomas were 119 ± 57 ng/mL and 258 ± 225 ng/mL, respectively. During menopause after treatment suspension, and at the latest follow-up: in microadenomas PRL levels were 23 ± 13 ng/mL and 16 ± 5.7 ng/mL, respectively; in macroadenomas, PRL levels were 20 ± 6.6 ng/mL ^**5t5**^and 25 ± 18 ng/mL, respectively. In menopause after treatment suspension, the microadenomas had disappeared in 9/22 and had decreased in 13/22. In the group of patients whose tumor had decreased, in the latest follow-up, tumors disappeared in 7/13 and remained unchanged in 6/13. In macroadenomas, after treatment suspension 3/7 had disappeared, 3/7 decreased and 1/7 remained unchanged. In the latest control in the 3 patients whose tumor decreased, disappeared in 1/3, decreased in 1/3 and there was no change in the remaining.

**Conclusions:**

Normal PRL levels and sustained reduction or disappearance of adenomas were achieved in most of patients, probably due to the decrease of estrogen levels. Dopamine agonists might be stopped after menopause in patients with prolactinomas.

## INTRODUCTION

The stimulatory role of estrogen on prolactin (PRL) secretion and on proliferation of lactotropic cells is well-established (1,2). Estradiol affects the secretion of prolactin at two levels: directly in the pituitary lactotroph, estradiol controls prolactin gene expression and modiﬁes its sensitivity to physiological stimulators and inhibitors of prolactin secretion; within the hypothalamus, estradiol modiﬁes the activity of the neuroendocrine neurons known to control prolactin secretion (3).

Menopause is a physiological condition of hypoestrogenism in which PRL levels are contradictory. Most studies showed a significant decrease while some others observed an increase in PRL levels in normoprolactinemic women (4-6).

There is scarce literature about the effects of menopause in patients with prolactinomas (7,8); we previously published a study about the effects of menopause in women with a history of microprolactinomas (9). The aim of this study was to retrospectively assess in the menopause the effects of the suspension of treatment on PRL levels and on the tumor size in patients with micro and macroprolactinomas diagnosed and treated with dopamine agonists (DA) during their fertile age.

## SUBJECTS AND METHODS

This is retrospective and multicenter study.

We studied 29 menopausal patients with prolactinomas diagnosed during their fertile age, 22 with microadenomas and 7 with macroadenomas. The macroadenomas were 5/7 intraselar and 2/7 had extraselar extension, the tumors dimensions ranged from 1.5 to 2.5 cm, without invasion of neighboring structures. The diagnosis was made based on PRL levels higher than the normal upper limit, symptoms as menstrual disorders and/or galactorrhea and the presence of a microadenoma or a macroadenoma in magnetic resonance imaging (MRI); furthermore the macroadenomas might present mass effect symptoms. All the patients had normal IGF-I levels according to their ages. This study was approved by the Ethical Committees from different hospitals participating in the Neuroendocrinology Department of Sociedad Argentina de Endocrinologia y Metabolismo. All patients gave written consent for their inclusion in this study.

The mean age at diagnosis of prolactinoma and at menopause was 40 ± 7.4 and 49 ± 3.6 years, respectively. There were no patients treated with hormone replacement during menopause.

Menopause was defined as the permanent cessation of menstruation resulting from loss of ovarian follicular activity after 12 months of amenorrhea following the final menstrual period, which reflects a near complete but natural diminution of ovarian function with no other obvious pathologic or physiologic cause (10). Elevated FSH levels confirmed menopause in our patients.

Hyperprolactinemia was defined as the presence of levels of PRL higher than reference range (> 24 ng/mL) in two or more times. In prolactinomas, we ruled out other pituitary pathologies with hyperprolactinemia when we observed a decrease in size or disappearance of the tumor and also the normalization of PRL levels in response to the treatment.

In 21/22 patients with microprolactinomas treatment was stopped when they reached menopause, only in one patient before menopause. In macroprolactinomas DA were stopped in menopause.

In menopause we stopped treatment in 28 patients whose PRL levels became normal (21 micro and 7 macroadenomas). The suspension of DA in one patient in her fertile age with a microprolactinoma was decided because the tumor decreased and PRL levels normalized.

No patients underwent any other treatment such as surgery or radiotherapy.

PRL levels were determined using different immunassays (IE) in each medical center involved in this study. Some IE were competitive design but most of them were not competitive. World Health Organization Standard 84/500 was the most frequently used.

PRL reference range for fertile women was below 24 ng/mL. The intra-assay and the inter-assay coefficients were lower than 2.7% and 6.3%, respectively.

MRIs were performed in all the centers and we considered Jules Hardy classification for pituitary neoplasm: microadenoma below 10 mm and macroadenomas higher than 10 mm.

The DA we used were bromocriptine (BEC) and cabergoline (CAB), with doses of 2.5-20.0 mg/day and 0.25-1.25 mg/week, respectively.

### Statistical analysis

The quantitative variables were performed with Shapiro-Wilk test. The parametric variables were expressed as mean ± standard deviation (mean±SD) and no parametric data as median (range). We used Studentand Wilcoxon test for the comparison of mean and median, respectively.

To assess PRL levels we calculated mean±SD and we used Student test to compare mean values. A p < 0.05 was considered statistically significant. The Statistical Analysis was performed with InfoStat software developed in Córdoba University, Argentina.

## RESULTS

Data were gathered at diagnosis before treatment, and throughout menopause when treatment was stopped and at the latest follow-up. Most patients were treated with CAB 18/29, five patients with BEC and 6/29 with both drugs in a consecutive way.

**Figure 1:**
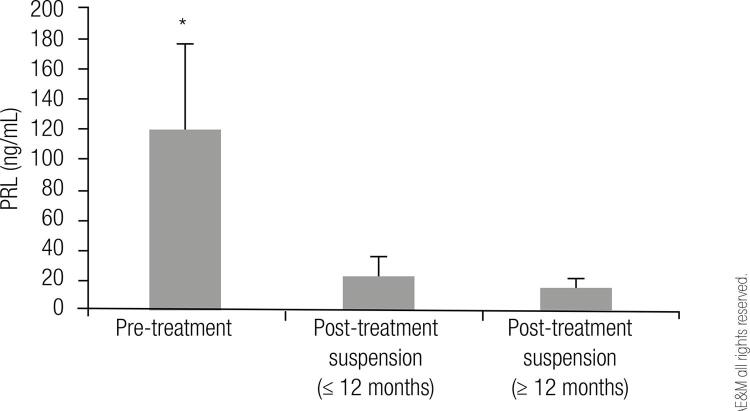
Prolactin levels before treatment and after treatment suspension in patients with microprolactinomas (* p < 0.001).

In microadenomas pre-treatment PRL levels during fertile age were 119 ± 57 ng/mL. During menopause, when treatment was stopped, between 4 and 12 months, PRL levels were 23 ± 13 ng/mL and at the latest follow-up, between 12 and 192 months after treatment suspension, PRL levels were 16 ± 5.7 ng/mL (Figure 1).

In macroadenomas, pre-treatment PRL levels were 258 ± 225 ng/mL and during menopause, when treatment was stopped, between 4 and 12 months, PRL levels were 20 ± 6.6 ng/mL and in the latest follow-up between 12 and 336 months after treatment suspension were 25 ± 18 ng/mL.

PRL levels at diagnosis in micro and macroadenomas were statistically significantly different when compared with levels after the treatment was stopped (p < 0.05), but there was no significant difference in PRL levels at suspension of DA and in the latest follow-up.

The average time of treatment was 135 ± 79 months. All the patients with prolactinomas under treatment normalized PRL levels.

Two years after suspension, 2 patients with microadenomas restarted treatment on their doctors’ decision, because of increased PRL levels similar to those at pretreatment.

**Figure 2:**
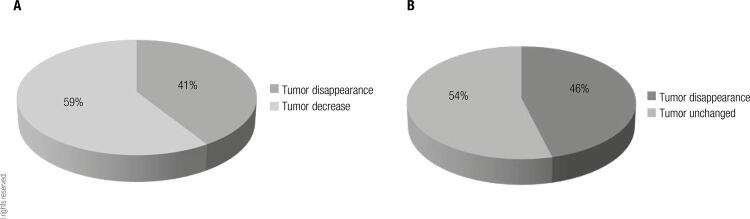
(A) Percentage of menopausal patients with microprolactinomas, in whom tumor had disappeared or had decreased when treatment was stopped. (B) Percentage of patients with microprolactinomas whose tumors disappeared spontaneously or remained unchanged in the latest follow-up, from the group in which tumors had decreased in A.

In fertile age, MRI images showed microadenoma in 22 and macroadenoma in 7 patients. In menopause after treatment suspension, in the subjects with microadenomas, the tumor had already disappeared in 9/22 and had decreased in 13/22 (Figure 2A). In the group of patients whose tumor had decreased, the tumor disappeared spontaneously in 7/13 and remained unchanged in 6/13in the latest follow-up (Figure 2B). In the patients with macroadenomas, after treatment suspension the tumor had disappeared in 3/7 (they have presented empty sella since then), decreased in 3/7 and remained unchanged in 1/7. In the latest control of the 3 patients whose tumor decreased, in 1/3 the tumor disappeared spontaneously, in another one the tumor decreased and in the remaining patient there was no change. In the patient whose tumor remained unchanged when DA were stopped, there was no change in the tumor size afterwards. In our patients, we observed that pre-treatment PRL levels did not correlate with the tumor size evolution in the menopause.

The time of follow-up of the patients from diagnosis to the latest assessment was from 9 to 33 years and after treatment suspension it was from 4 to 192 months.

## DISCUSSION

PRL levels in women of reproductive age are higher than in menopausal women because of higher estrogen levels (11). Menopause seems to have a beneficial effect on the natural history of hyperprolactinemia because of the declining estrogens levels that accompany the cessation of menses.

The medical treatment of hyperprolactinemia is mainly based on DA use. The response to these drugs varies; approximately 95% of patients treated with standard doses reach normal PRL levels with the tumor decreasing in size or disappearing. Prolactinomas diagnosed in postmenopausal women usually display responsiveness to DA too (12).

The DA available in Argentina are BEC and CAB, the latter being more frequently used because it has more affinity to the dopamine receptor, fewer side effects, and is more effective than BEC (13,14).

In this retrospective study, in 90% of patients with microadenomas whose treatment with DA was stopped during menopause experienced tumor reduction in size and maintained PRL levels within the normal range. Additionally, the patient with microprolactinoma whose treatment was stopped before menopause maintained normal PRL levels throughout menopause. In patients with macroprolactinomas the PRL levels after treatment suspension were in the normal range; and in the latest control some patients, including the three patients with tumor persistence, showed slightly increased levels above the upper normal limit, this situation might be due to the effects of menopause.

Most of the prolactinomas are microadenomas (15,16) and this was also observed in this study. In prolactinomas there is a close relation between PRL levels and the tumor size; the bigger the tumor is, the higher the PRL levels are. It is not frequently observed that a tumor becomes bigger without increasing PRL levels (15). In our study, we observed that the tumor size evolution in menopause did not correlate with the pre-treatment PRL levels; there were patients with highly increased levels who showed tumor disappearance in their images, while some others with lower levels presented only some decrease in the tumor size. It is difficult to explain this situation if we consider that the mechanisms involved in the possible relationship between PRL transcription, PRL levels and lactotrophs proliferation are not clear (17). Estrogen and dopamine are major opposing regulators of the functions of lactorophs. Dopamine, through the D2 receptor (D2Rs) in lactotropes, inhibits cell proliferation and prolactin secretion (18,19). The therapy with DA is effective in most patients, but it is described that approximately 15% of them are resistant to these drugs. A decrease in number or function of D2Rs would be the cause of the discordance observed between PRL levels and tumor size (20).

In our patients with microaprolactinomas, the tumor disappeared spontaneously during menopause in about 50%. In patients with macroprolactinomas, during menopause, one out of the three tumors which had decreased disappeared spontaneously. These outcomes are probably due to the effect of menopause.

Touraine and cols. in their retrospective study assessed the role of estrogen in women with a history of hyperprolactinemia and found that in a group of untreated patients, PRL levels decreased spontaneously during menopause (8). At the current time there is a lack of evidence to advocate the treatment of asymptomatic postmenopausal women with prolactinomas (21,22). Chanson and cols. suggest that treatment with DA may be discontinued after menopause in microprolactinoma because of two reasons, the first being the lack of evidence of harmful effects of hyperprolactinemia on health other than on gonadotropic function, especially since there are no convincing epidemiological studies in favor of an association between hyperprolactinemia and breast cancer, and the second being spontaneous gradual normalization commonly seen in PRL levels after menopause, 44% of cases in one study (7,23).

In this retrospective study with a long follow-up of patients with a history of hyperprolactinemia whose treatment with DA was stopped when they reached menopause, PRL levels normalized or decreased probably due to the fact that estrogen levels diminished. However, two patients with microprolactinoma restarted treatment on their doctors’ decision when their PRL levels increased two years after the suspension of DA.

In conclusion, the importance of these observations might justify the suspension of DA treatment at menopause in patients with micro and macroprolactinomas diagnosed during their fertile age.
